# Biomimetics: teaching the tools of the trade

**DOI:** 10.1002/2211-5463.12963

**Published:** 2020-09-28

**Authors:** Kristina Wanieck, Daniel Ritzinger, Cordt Zollfrank, Shoshanah Jacobs

**Affiliations:** ^1^ Working Group Biomimetics THD—Technische Hochschule Deggendorf (Deggendorf Institute of Technology) Freyung Germany; ^2^ Biogenic Polymers TUM Campus Straubing for Biotechnology and Sustainability Technische Universität München Straubing Germany; ^3^ Faculty of Applied Natural Sciences and Industrial Engineering THD ‐ Technische Hochschule Deggendorf Deggendorf Germany; ^4^ Department of Integrative Biology and Office of Educational Scholarship and Practice University of Guelph Canada

**Keywords:** biologically inspired design, biomimetics, biomimicry, education, teaching, training

## Abstract

Biomimetics is a known innovation paradigm of the twenty‐first century with significant impact on science, society, economy, and challenges of sustainability. As such, it can be understood as a mindset for creative thinking and as a methodology or technique for effective knowledge transfer between disciplines, mainly biology and technology. As biomimetics is relevant to practitioners in various fields of application, understanding the teaching and training of biomimetics for different audiences is important. With this article, we aim to give a holistic view of teaching and training practices and opportunities. First, we offer a set of learning objectives based on an analysis of various courses worldwide and we give recommendations for the design of future curricula. Second, based on an audience analysis and interviews, we developed a set of personas of the users of biomimetics, and as such, we offer a deeper understanding of their needs for the design of the process, including tools and methods.

## Graphical abstract

Effective teaching requires an understanding of the student learning context. These ‘personas’ can then be matched with appropriate learning outcomes to design meaningful experiences that will support the student in applying their new skills. Here, we analyze the personas engaged in biologically inspired design (BID) and the associated learning outcomes. Recommendations for teaching BID are included.

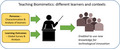

**Abbreviation**
BID: biologically inspired design

Whenever research and development addresses learning from nature, several approaches can be responsible for the outcome, represented by various terms and definitions [1]. In addition, the abstraction and application of nature's principles and strategies to solve practical problems ranges from inspiration (e.g., shapes for industrial design), to knowledge transfer and emulation (e.g., hook and loop attachments), to scientific implementations (e.g., biomimetic materials). The level of abstraction gets higher with increasing depth of research, understanding of biological principles and in the requirements for abstraction, transfer, and implementation [2]. Though learning from nature has a long history in humankind and in science more specifically [3, 4, 5], it remains controversial whether it is still in its infancy [6] or whether it can be considered established in technology [7, 8]. Nevertheless, as with any new development or innovation that is inspired by nature it starts with an individual having a new idea. Therefore, this manuscript focuses on the audience of biomimetics, that is, the users who are taught in the topic and shall be enabled to actually doing biomimetics in practice.

One key aspect in performing knowledge transfer from nature is the how‐to. The ideas and assumptions of biomimetic practice sound appealing: nature's biological systems have faced the same problems as engineers' technological systems because we share a common environment. To ‘solve’ these problems, nature has evolved through adaptation over billions of years' time, and therefore, the animate nature that we observe today in millions of species can be—in theory—the realm of solutions available to solve current engineering problems or improving technological systems. The impact of learning from nature can be in science, technology, economy, society at large and sustainability [3, 9]. However, the process, methodology, deeper understanding and implementation of nature's principles in practice remain difficult to conceptualize. The topic is complex [10], and doing it is, often by definition, an inter‐ and transdisciplinary process. Learning from nature is a promising paradigm for innovation and sustainable design, and 'the scientific challenge now is to transform it into a repeatable and scalable methodology' [11]. One big challenge in this transformation is to ‘educate new generations of would‐be‐designers in the paradigm of biologically inspired design’ (BID), as postulated by Goel *et al*. [11]. In order to achieve this, it is important to understand future designers, to define the paradigm of BID with its content for teaching and to define clear objectives of teaching. As the field of BID is very broad and combines multiple disciplines, we consider it helpful to provide an overview of various aspects in the context of education in BID.

A first step in this transformation, as presented in this manuscript, focuses on learning the methodology, based on its teaching and training. Therefore, we review and analyze the state of education within formal and private programs, and address the question of different audiences in higher education and practice, who have different learning outcomes and a different set of good practices. Based on an audience analysis and interviews, we make suggestions for tailored curricula matched with the needs of users. A second step in this transformation is linked to the methodology in theory and practice. To contribute to the development of a repeatable methodology, we present data on tools linked to the process, which can support the content of curricula.

## Terminology

As an inter‐ and transdisciplinary topic, several perspectives exist about what learning from nature actually is (see, e.g., [1]), how it should be performed, and how it may develop [3, 7, 12, 13]. This paper represents one such view and intends to contribute to the field.

The term biomimetics is used in this manuscript because it is most often used in describing its scientific history and impact [3, 4, 5, 9, 14], and its process [1, 2, 15, 16, 17, 18]. It has also gained international acceptance, represented by the International Standard on Biomimetics [8]. Furthermore, as per the definition, it can be used to solve practical problems through an ‘interdisciplinary cooperation of biology and technology or other fields of innovation’, using ‘the function analysis of biological systems, their abstraction into models, and the transfer into and application of these models to the solution’ [8]. As such, biomimetics can combine various disciplines (e.g., natural and engineering sciences, product development, innovation, design) and can impact several fields of application, leading to various subfields of biomimetics. In addition, depending on the depth of application, biomimetics can be considered to be a scientific discipline, an innovation process, or a creativity technique [8]. As such, this manuscript focuses on biomimetics and intends to support its understanding and implementation.

However, though the definition and application of biomimetics is broad, it is restricted mostly to its research quality, as the International standard states: ‘Biomimetics is founded on fundamental research in the field of biology. Due to its defined focus on applications, though, it primarily integrates application‐oriented and applied research into the actual development of the product or process’ [8]. Furthermore, its scientific orientation becomes obvious through its differentiation from other approaches, which are defined as ‘creative approach’ (bio inspiration), ‘philosophy’ or ‘interdisciplinary design’ (biomimicry, biomimetism) [8].

Therefore, the wider term BID will be used in this manuscript, too, in order to include other approaches, which focus on transferring principles from nature to engineering design through the abstraction of biological principles and the transposition to technology or practice, in general. In some cases, the respective approach can be departed from the need of fundamental research, for example, once it is used for the generation of new ideas. In this context, biomimetics can be:

1. A scientific discipline of fundamental research for knowledge transfer between biology and technology (biomimetics) [3, 8].
  Example: Development of biomimetic materials, for example, dry adhesives inspired by the gecko [19].
2. A problem‐solving strategy as part of the overall product development process (top down process of biomimetics, [2, 8, 20]).
  Example: A self‐sharpening knife [21].
3. A means to develop new ideas and products (as part of 2.), or for rethinking a product (blue ocean strategy, bottom‐up) as a creativity technique or a systematic approach, based on catalogs or biological principles.
  Example: Biological materials with special mechanical properties to inspire biomimetic devices [22].
4. A means for sustainable solutions [1, 12, 23, 24, 25], for example, based on material and energy efficient systems.
  Example: Material efficient structures through Soft Kill Option [26].
5. An innovation strategy [27].
  Example: Bionic Learning Network, FESTO AG & Co. KG [28].

Teaching will refer to any curriculum, learning objective, and teaching environment based in academia. The audience for this will be referred to as students. If necessary, the graduate level will be distinguished.

Training refers to any education of professionals from the business sector, representing the second main audience and target group for the application of biomimetics. It will also address experts, for example, from natural sciences (mainly biology) who have a degree and would like to use their knowledge for biomimetics or BID.

## Education and audiences

The knowledge transfer from biology to technology or other fields of application requires a deep understanding of the process of biomimetics [2, 29] that is further demanding because of the diversity of disciplines from which relevant tools have been developed [2, 29, 30, 31]. The field of biomimetics brings together a diversity of people, motivated to achieve a variety of outcomes. Their teaching and training are crucial for the success of its implementation in practice and for the increase of existing products as proof of concept. However, they are taught and trained in very different environments, as settings of learning, teaching, and using knowledge in academia tend to be very different from work settings [32, 33]. While students acquire knowledge for future usage, practitioners need to solve problems with respect to their possibilities and boundaries set by the entity they work for. Also, from our experience in teaching students, in training industry, and in informing society at large, we hear from users that after taking the training offered, they still feel disconnected and unable to transfer the concepts to their specific context. Therefore, we consider an analysis of the existing educational content to be of interest for the transformation into a repeatable methodology.

Any methodology facilitating the biomimetic design process needs to fit to its focus and for this reason, the steps may differ drastically depending on, for example, the aim of use [6]. Additionally, even though the user of biomimetics could be a single individual, in general, product development is an interdisciplinary venture [34]. Interdisciplinarity in biomimetics is a key aspect [35, 36, 37, 38, 39, 40], which we consider to go beyond this manuscript, and therefore, it is not addressed here in particular. Biomimetic projects vary highly depending on the topic and the disciplines involved. In order to draw conclusions from our work for general recommendations, we make some rough generalizations, for instance of users, like a practitioner, or a student. As we did not consider individual personal backgrounds, we add aspects of an audience analysis and defined an initial set of personas, in order to clarify needs and expectations of various users. We intentionally do not consider a differentiation between novices and experienced users (see, e.g., [41, 42, 43]), nor cognitive aspects, which have been successfully addressed in other research (e.g., [44, 45, 46, 47, 48, 49, 50]).

Analyzing the learner audience and their learning context is essential whenever information is transferred through written, spoken, or visualized communication [51], such as in technical communication or in teaching practices. The better you know your audience, the more appropriate will be the knowledge translation and the more effective will be the knowledge transfer. In the context of biomimetics, the target audience groups are expected to implement their newly gained knowledge for, for example, product development and design. However, common challenges regarding the tasks of the process are well known [35, 44, 45, 48, 49, 52, 53] and have yet to be overcome. Furthermore, though a large knowledge base in biomimetics is accessible [3], the number of real products on the market is manageable [54]. We consider an audience analysis to be a step toward bridging the gap of a present limited implementation and a better understanding of the current situation in education. Here, we conduct an analysis of the existing learning objectives, the learning contexts, and the types of learners (personas) associated with BID. We combine our findings to make recommendations on teaching gaps, and developing appropriate curriculum for target audiences [55, 56].

## Materials and Methods

### Literature survey on existing curricula

#### Topics of teaching modules

The scientific literature was screened for teaching and training settings of biomimetics and related fields. Search terms included ‘teaching’, ‘training’, ‘education’, ‘course’, ‘class’, ‘BID’, ‘biomimicry’, ‘biomimetics’, and combinations of the terms. As there are various overviews and analyses on teaching institutions and practices, for example, [40, 57], the search was also based on the references sections in identified relevant publications.

The aim of the literature survey was to identify and compare learning contexts, objectives, experiences, and audiences in teaching and training of BID. Therefore, the literature survey focused on scientific literature, rather than simply establishing a list of institutions teaching and training the topic. Different reports exist which present more than 100 educational endeavors in the context of biomimetics, biomimicry, or related topics, for instance in Europe and North America [58, 59]. Such a list can hardly ever be exhaustive as it would need research of the existing curriculum of each program at all higher education institutions. Such an analysis was not the focus of this manuscript. A limitation to this survey is that experts and leaders in the field are not listed, even though their work is well known. However, the authors consider such a list at any stage informative and representative as it provides insights on (a) the international distribution of the topic, (b) the increasing interest in educating students, and (c) the variety and diversity of teaching opportunities. Therefore, institutions from such reports were added once they offered information on the topics of interest for this manuscript, even though their information was only documented on their websites. In such cases, the websites were the only source to refer to and they were added, for example, when they gave insights into the respective learning objectives. The learning objectives of the classes of the first author were also included, in order to share own work.

As a result, the overviews presented in this manuscript are excerpts of existing teaching practices and differences as described in scientific literature or on websites that offer up‐to‐date information. The scientific literature might reflect the specific research interests of the authors rather than the depth of the teaching practices. However, with this in mind, the results are based on the analysis of published data and from which it was possible to draw conclusions.

#### Learning objectives

**Fig. 1:**
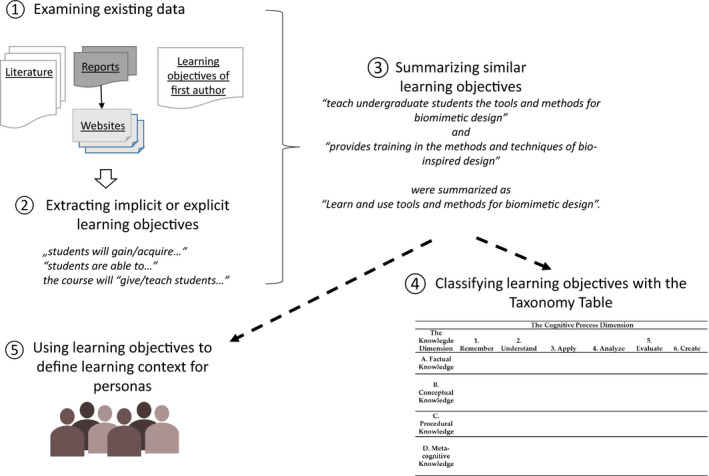
Qualitative research method to classify main learning objectives.

The learning objectives were extracted from the respective literature or from websites which offer a detailed description of their academic programs or courses. Learning objectives can be written implicitly or explicitly. The list refers to those objectives that were mentioned explicitly as learning objectives or implicitly as learning emphasis, like ‘students will gain/acquire…’, ‘students are able to…’, or the course will ‘give/teach students…’. Similar objectives were summarized to the presented list (Table 2), like ‘teach undergraduate students the tools and methods for biomimetic design’ [31] and ‘provides training in the methods and techniques of bio‐inspired design’ [40], which were summarized as objective 8 ‘Learn and use tools and methods for biomimetic design’. Figure 1 summarizes the methodological approach to deduct and classify learning objectives.

#### Taxonomy table

In order to analyze the current teaching practice represented by the identified learning objectives, we used the Taxonomy Table as described by Krathwohl [60], which is based on Bloom's taxonomy. The Taxonomy Table offers the possibility to examine the emphasis of teaching and classifies learning objectives based on their reference to the Knowledge Dimension (A. Factual, B. Conceptual, C. Procedural, D. Meta‐cognitive) and the Cognitive Process Dimension (1. Remember, 2. Understand, 3. Apply, 4. Analyze, 5. Evaluate, 6. Create) [60]. In the case of an interference of one objective with more than one dimension, we selected the deepest of the table dimensions understanding that they could apply upward. For instance, objective 1 ‘Have a solid understanding of BID with its various approaches (differences and similarities)’ refers to understanding factual knowledge (A2) and the conceptual (B2). Therefore, we chose the deepest.

Or objective 9 ‘Assess lifelong problems using BID by developing creative, innovative ideas and solutions’ refers to analyzing the procedural knowledge dimension (C4), but also the application of conceptual knowledge (B3).

### Research methods for defining learning contexts for BID personas

A combination of research methods, described below, was used for the derivation of the relevant personas and scenarios. The persona represents a prototype of a group of users with, amongst others, detailed characteristics, specific goals, and needs of information [61]. As the development and definition of persona have their challenges, we strengthen our analysis with aspects of a data‐driven development [62], specifically qualitative observations from the identified courses, and a complementary analysis of interviews with practitioners.

#### Interviews and survey for personas

First, the BioM Innovation Database (*n* = 379) and associated interviews with inventors of the respective products were analyzed [54]. The data collected by these interviews served to provide insight on the process of product design, the level of disciplinarity, and the understanding of biology of the design team. It represents a complementary collection of information to support the BioM Innovation Database. The BioM Innovation Database was gradually filtered through our focus on certain topics. One limitation of the BioM Innovation Database is incomplete data because questions remained answered with N/A, due to confidentiality or lack of information available or given in interviews. Therefore, we filtered and categorized data for a comparable analysis, including those cases that offer answers to the relevant research question. For example, when we analyzed which disciplines were involved in the development processes we analyzed the cases that offered answers to that question. Cases not giving insight to this question were not considered. Therefore, various results refer to subsets of the *n* = 379 cases of the BioM Innovation Database.

Second, surveys with 14 practitioners of biomimetics were analyzed. The surveys were sent out to practitioners in Germany, who worked at companies that either have (a) a biomimetics‐derived product on the market, (b) a biomimetics‐derived product which is in development or in prototype phase, or (c) participated in a training or workshop on biomimetics and have firsthand experiences with the topic. Out of the 21 invited practitioners, 14 responded. The survey encompassed 30 open or closed questions and was conducted to understand the practitioners' needs and interests in the broad context of biomimetics. It had five parts giving information on (a) general information of the company, (b) endeavors on systematic innovation, (c) interdisciplinarity and networks of innovation, (d) biomimetics, and (e) sustainability.

Even though the sample size is small, we consider the results to be of value because of the level of expertise of the participants that enabled us to first identify initially nine different personas. In addition, we cross‐checked our initial personas with real‐life situations of 24 taught classes, 12 interviews with experts from industry and academia and nine workshops in which we trained industry in biomimetics over a 9‐year period. In the end, the personas resonated with the feedback and discussions of more than 300 engineering students, more than 320 (innovation) management students and 18 CEOs and project leaders in industry who we have met, taught, and engaged with BID personally. Using purposive sampling (sampling which target‐specific experts), we contacted peers with direct knowledge and experience in BID and asked them to review the personas list. Their suggestions were included.

**Fig. 2:**
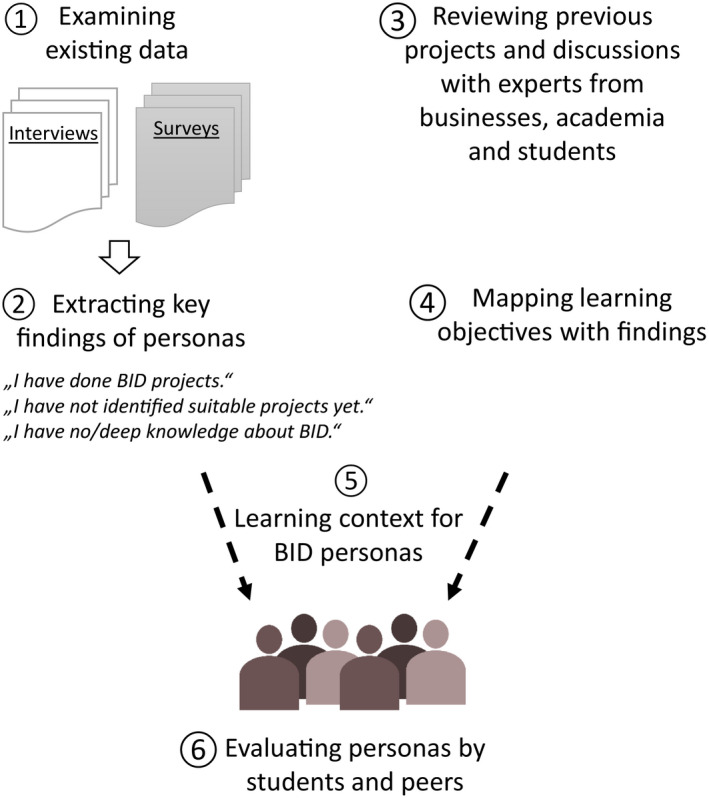
Qualitative research method to define the learning context for BID personas.

To verify the personas we conducted a survey with 20 students from a Master's class in Innovation and Product management in fall 2018 at the University of Applied Sciences Upper Austria who were taught in BID by the first author (KW). The students were asked to identify learning objectives from Table 2 which they consider to be of relevance for them, whether the persona fit to their personal situation, and whether they were interested in inspirational talks or specific training in BID. Figure 2 summarizes the research method to define the learning context for personas.

All protocols were approved by the Research Ethics Board under certificate number 12OC062 from The University of Guelph. All participants gave consent either in written or electronic form. All data were anonymized after the collection was complete.

## Results and Discussion

### Literature review on teaching and training methodologies

**Fig. 3:**
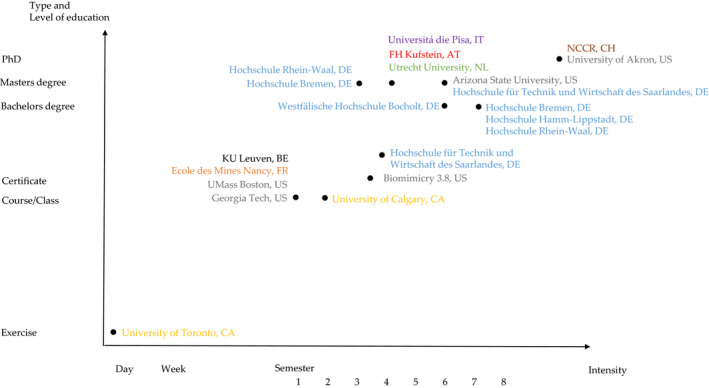
Intensity of teaching practices at various institutions. The colors differentiate the country, where the institutions are located in Europe (Austria, red; Belgium, black; Germany, blue; Italy, violet; Netherlands, green; Switzerland, brown) or North America (Canada, yellow; United States of America, gray).

The literature and report review identified 29 universities or institutions worldwide that teach and train BID students and/or professionals and have published data on their programs or classes. Several teachers assessed the effectiveness of their course and published the data about cognitive or pedagogical aspects of the teaching. In general, there are two distinct ways of engaging with a BID curriculum as a student (see also Fig. 3):

1. Studying programs for a Bachelor's, Master's, or PhD degree in BID related topics, and
2. Learning BID as an additional qualification alongside a disciplinary program in natural, engineering, or other sciences.

#### Topics of teaching and training BID

Four main topics encompass teaching and training in BID currently: Biomimetics, biomimicry, BID, or bionics, with partly specific focuses, for example, robotics, architecture, or innovation (Table 1).

**Table 1:** Different topics of BID taught in academia. The asterisk stands for any linked topic with ‘bio‐inspired’, like amongst others design, engineering, or materials.

Topic	Title of the learning opportunity	References
Biomimetics	Biomimetics	[20, 63]
	Biomimetic design	[31, 42]
	Biomachines and Biomimetics	[64]
	Biomimetics Motion Systems	[65]
	Construction biomimetics	[66]
Biomimicry	Biomimicry	[55, 67, 68, 69]
	Biomimicry for Designers	[70]
	Biomimicry: From life to nanotechnological innovations	[56]
	Biomimicry in Architecture	[71]
Bio‐inspired*	BID	[37, 49, 53, 72, 73]
	BID of sport	[74]
	Bio‐inspired engineering	[52]
	Bio‐inspired innovation	[75]
	Bio‐inspired materials	[76]
	Bio‐inspired robotics	[77, 78, 79]
Bionics	Bionics	[80]
	Bionics engineering	[81]
	Bionics/biomimetics	[82]
	Material Design—Bionics and Photonics	[83]
	Science communication and bionics	[84]
Others	Bio‐TRIZ	[85]
	Integrating biology and design for sustainable innovation	[40]
	Design Innovation from Nature	[86]^a^

^a^ Studio One is an alternative teaching model, which focused on the topic of Bio‐inspired Design and Fabrication for the academic year 2016–2017 and 2017–2018. It is listed here as well to show that BID can be a means to facilitate specific teaching practices.

The variety of topics taught in the context of BID (Table 1) further demonstrates how important the clear definition and understanding of biomimetics and related topics is. Especially, as the terms are commonly used as synonyms or to address, for instance, the scope of a journal's issue. Therefore, we suggest to present and explain the approaches as introductory and central content in teaching modules so that future generations of users are aware of the definitions, differences, and synergies. The international standard ISO 18458 [8] is the most advanced document to define different approaches and concepts and to describe the methodology of biomimetics. Some terms are missing in the standard, like ‘biologically inspired innovation’ [75], and it is likely that more permutations of the terms will arise in the future, as it is already happening with, for instance, ‘biological transformation’ [87]. However, the standard provides a framework which will evolve in the future and for which there will be a documented history.

#### Intensity of teaching practices and geographical distribution

The teaching of BID ranges from a single exercise or module, seminar or workshop, to a class or course, up to Bachelor's and Master's degree, or a PhD program, with a duration from 20 min to 5 years. The highest level of education is a PhD program offered in both the United States and Europe. Figure 2 visualizes the variety of teaching practices at 19 institutions, and it reflects the above mentioned two distinct ways of engaging with BID in a curriculum.

That the institutions presented in Fig. 3 are dispersed geographically across Europe and North America, presents an important determinant of curricular variation across the programs because there are geographical differences in the understanding of and the motivation for BID. Three of the identified countries focus on biomimetics and related topics, five on BID, two on biomimicry, and two on bionics. For instance, France and the Netherlands focus on sustainability‐driven BID (e.g., [75, 88]), while Germany focuses more on the technological developments of biomimetics, as represented by the existing Bachelor's and Master's degrees.

#### Learning objectives of teaching practices

Comparing current curricula and learning objectives, we found that teaching content varies in accordance with, for example, the universities' or professors' research focus or the background of the program, emphasizing that there are various audiences and associated learning outcomes. Based on the literature review, we present 18 primary learning objectives of teaching BID, as summarized in Table 2.

**Table 2:** Primary learning objectives for teaching BID.

	Learning objectives	References
Objective 1	Have a solid understanding of BID with its various approaches (differences and similarities)	[69]^a^
Objective 2	Have a more complete knowledge base	[77]
Objective 3	Use their own expertise for BID	^a^
Objective 4	Find an individual access to the topic (through various motivations, like sustainability, innovation management, engineering, creativity)	^a^
Objective 5	Discover and Explore Nature's patterns, strategies, and solutions for analogous problems	[64, 89]^a^
Objective 6	Learn the conceptual process leading to bio‐inspired design	[74]^a^
Objective 7	Learn the procedural process leading to bio‐inspired design	^a^
Objective 8	Learn and use tools and methods for biomimetic design	[31, 40, 74, 90]^a^
Objective 9	Assess lifelong problems using BID by developing creative, innovative ideas and solutions (novel and diverse; eventually sustainable and resilient)	[69, 72]^a^
Objective 10	Plan and manage BID projects	^a^
Objective 11	Develop a biomimetic mindset	[69]
Objective 12	Develop their integrative skills	[20, 90]
Objective 13	Develop cognitive flexibility	[46, 53, 57, 90]
Objective 14	Train creativity	[46, 53, 57]^a^
Objective 15	Develop adaptive problem‐solving skills	[46, 53, 57]
Objective 16	Have cross‐disciplinary thinking	[46, 53, 57]
Objective 17	Work, engage, communicate and collaborate across disciplinary boundaries and in interdisciplinary teams	[20, 40, 89]^a^
Objective 18	Explain the topic, working principles and fabrication methods to a variety of audiences	[64, 69]

^a^ Objectives in classes of first author

The overview of learning objectives from education and training shows that these objectives address overarching topics, like (a) personal motivation and development (objective 3, 4, 11), (b) a solid understanding of the BID development process, its foundation and application (objective 1, 2, 5, 6, 7, 8), (c) skills that are an outcome of working with BID but aren't specifically about learning BID (objective 12–17), and (d) a future expertise that is gained (objective 9, 18). As such, BID can be a tool for interdisciplinary education [37].

In order to help design future curricula, to serve various audiences, and to overcome well‐known challenges in learning how to actually do BID, we analyzed the current teaching practice by using the Taxonomy Table [60], based on Bloom's taxonomy. The Taxonomy Table offers the possibility to examine the emphasis of teaching, to classify objectives, to evaluate the extent of higher order learning, and to align and plan curricula by identifying missed educational opportunities [60]. In Table 3, the identified 18 learning objectives are classified in the Taxonomy Table.

**Table 3:** Main learning objectives for teaching BID classified with the Taxonomy Table [60]. The green color indicates objectives that are often referred to in inspirational teaching without curriculum to teach them, while all objectives might be addressed in training curriculum.

The Knowledge dimension	The Cognitive Process Dimension					
	1. Remember	2. Understand	3. Apply	4. Analyze	5. Evaluate	6. Create
A. Factual knowledge						
B. Conceptual knowledge		Objective 1				
		Objective 2				
		Objective 5				
		Objective 6				
		Objective 8				
C. Procedural knowledge		Objective 7^a^	Objective 8	Objective 9		Objective 10^a^
						Objective 12
						Objective 15
D. Meta‐cognitive knowledge		Objective 18	Objective 17	Objective 3^a^	Objective 4^a^	Objective 11
						Objective 13
						Objective 14
						Objective 16

^a^ Objectives unique to courses taught by first author.

Table 3 visualizes a clear emphasis in teaching BID on Understanding the concept and on Creating solutions. All of the defined objectives involve cognitive processes that are considered ‘higher order’ [60]. On the other hand, this result highlights the difficulty of teaching and learning BID. The topics are complex and ask the students to involve their cognitive skills at a higher level. As such, teaching should differentiate between an inspirational talk and a specific training. Therefore, we divided the objectives along these two categories. All learning objectives can be relevant for training and deeper understanding, while we classified eight objectives relevant for inspirational talks (colored green in Table 3). An inspirational talk is used to promote the topic without giving real insight into the process or its practice. Though many of the learning objectives are referred to in these presentations, there is no curriculum associated with them. Therefore, the addressed objectives do not necessarily need depth of knowledge. Interestingly, these eight objectives refer mostly to the meta‐cognitive knowledge dimension (six out of eight), and to the conceptual knowledge (two out of eight). The objective can be addressed in detail and greater depth, and as such, they become relevant for training, though.

Additionally, Table 3 indicates that column 1 ‘Remember’ remains empty, and line A ‘Factual Knowledge’ contains only one objective. This could be because that each BID project or problem to solve is different and has its own specificities so that there is no blueprint for factual knowledge that needs to be learned and applied repeatable. Rather the conceptual and procedural knowledge need to be adapted for each project, which again highlights the high demands toward learning BID.

As the focus of the education is skills‐based, the academic framework to facilitate interdisciplinary learning environments could adapt [91]. In addition, ‘the Taxonomy Table suggests what might have been but wasn't’ [60], as the blank spaces indicate that there might be some more teaching opportunities which have been underrepresented so far. These could include, for instance,

1. Evaluation and assessment of problems (A5, B5)
  In ongoing research, we focus on the assessment of problems that can be addressed with BID, especially from the perspective of a practitioner. This is a crucial question to address prior to starting a project in order to save money, time, and energy.
2. Evaluation of successful steps (C5)
  As the process of BID is iterative and involves various channels of knowledge, students could evaluate the successes during the respective steps of the process with greater intensity. One of the key challenges mentioned in the literature is the abstraction and 1 : 1 transfer [35], and if evaluation steps are included, the learning outcome could be more effectively achieved.
3. Evaluation and assessment of solutions (B5, C5)
  Various endeavors have focused on classifying the outcome of BID with regard to, for example, being biomimetic or nonbiomimetic [8], to differentiate several types of outcome [1], or the assessment of sustainability [23, 92]. In addition, the ISO TC266 has worked on an assessment to evaluate the process and innovative potential of the BID process (unpublished). Our ongoing research is also focused on this aspect, and it could be a valuable contribution to the teaching and training of BID.

Most of the curricula include exercises, activities, or workshops in natural settings. These activities are considered to be very important in order to assist students with applying the factual and conceptual knowledge (A3, B3). In addition, they support the educational research on the importance of immersive experiences [93, 94]. An important aspect here is, that ‘tell and practice’ methods of teaching are not enough, as it leaves the student unable to transfer the newly gained knowledge to a different context [95, 96]. One source mentioned a ‘team teaching’ approach that is supported by an interdisciplinary teaching team, so that the course format required a high engagement of students and led to the improvement of student–teacher relationships [40]. The learning objectives indicate various contents that need to be addressed in teaching modules. Therefore, we analyzed the actual content that is taught.

#### Structure and content of courses

The structure and content of 12 identified courses and classes were analyzed. These courses were chosen as they are described in detail in the literature or on the website of the respective institution. Besides unique elements, like, for example, a photo journaling activity [40], there is a larger pattern in what is taught in biomimetics or BID. The common themes and emphasis are:

1. Theory
  Background knowledge about the overall topic or specific subtopics.
2. Tools
  A means to be used in performing or supporting the biomimetic process or parts thereof.
3. Case studies
  Success stories, classic products, or examples of implementing BID.
4. Methodology
  The overall process and approach in its details.
5. Exercises
  Hands‐on exercises performed by the students to explore nature or subtopics of BID.
6. Implementation
  Design challenges or exercises which guide students through the overall process from a problem to a BID.

Differentiation between inspiring and training audiences can be of help to tailor educational approaches. The presentation of theory and case studies is appropriate and sufficient for inspiration. But when the goal is to teach and train, the methodology, tools, detailed knowledge about the process, exercises, and implementation challenges should be included.

### Learning context for BID personas

#### Information from the literature

The research of existing curricula suggested that they have been designed to target various learning contexts. In general, there are several ways of enabling users to consider BID in practice which are executed at present as:

1. Information and inspiration
  Inform various groups of interest about the topic via inspirational talks, promoting the topic or giving a first glance. Often, the audience is an in‐the‐loop‐audience, as they will not use the knowledge necessarily. However, they need to know about BID as they might be from funding agencies, the general public, or target groups connected somehow with the topic, like NGOs. Another aim of such a talk could be for example, to engage representatives from industry. In this case, they become an actioning audience, which acts on the information provided. They would be the priority audience and they need the information to implement it in their own context. One of the biggest challenges then is to connect research and practice and to find, for example, project partners.
2. Teaching
  Teaching biomimetics is directed toward an actioning audience.
  - Middle and secondary school students: To inform and teach students at school is not considered in this manuscript. However, it has been of interest in research, for instance in the development of educational material [97], and supports the notion of starting to introduce these topics early in a person's formal education.
  - Postsecondary students: One of the most important target groups for learning and implementing biomimetics and BID are students in postsecondary academia, as they focus their studies toward future career pathways. Therefore, there are various teaching opportunities within formal institutions including a single class, a full course, or a full program, for undergraduates or graduate students. As such, students either gain additional expertise or a distinct credential such as a degree (see also [53, 57]).
3. Training
  The second most important target group for learning BID is the group of professionals predominantly from the business sector. Once inspired, as mentioned in 1, they can be trained as an actioning audience in workshops or online courses to earn postgraduate certificates or diplomas. They can be trained at private institutions or in academia, and of course, they might then be considered as student again, once they, for instance, subscribe to an extra‐occupational program of study (a.k.a continuing education). For the sake of completeness, there is also the aspect of supporting professionals by consulting services, which is not considered in this manuscript as it is proprietary to the respective firm.

To sum it up, the word ‘student’ applies to many different types of people, from those who have previous training (practitioner), to those without previous knowledge (undergraduates), from those with general curiosity to those with specific intentions to use BID. As such, they have different needs and expectations that require different curricula. To find out more about the various personas interested and involved in BID, we analyzed 73 interviews (14 practitioners, 59 BioM database cases).

#### Interviews

The interviews with 14 practitioners showed that 36% of the companies were small and medium sized with 50–249 employees, and 64% were large with more than 250 employees. Each of the interviewed companies identified themselves as being open to collaborate with external expertise. 21% were in constant exchange with third parties, 36% regularly in projects, and 43% from time to time. Interestingly, 21% of the companies had not yet identified a suitable project for BID, though they had a certain experience or familiarity with the topic.

When asked about internal factors which support or hinder the success of BID for the company, 57% said that their employees are not at all, mostly not, or in parts not familiar with BID as a problem‐solving strategy, reflecting the need to have a scalable methodology. The interviewed persons usually considered BID an ‘inspiration’ or ‘learning from nature’.

57% of the companies could not tell whether they would employ a person who had studied BID, as they did not know their expertise well enough, though 43% consider it as beneficial to have someone on the team who has experience in BID. Currently, in Germany, there is a large‐scale Delphi study underway to analyze the fields of occupation for BID, which will help frame the expertise and usefulness of BID‐educated people.

Based on the interviews combined with the information from the scientific literature and from our experiences in teaching and training various audiences in biomimetics, personas were derived in the context of BID. Table 4 shows 10 different personas that are described briefly.

**Table 4:** Personas in the context of BID and matching learning objectives.

Company background	Characteristics	Objectives
BID Enthusiast	Is fascinated about the topic Does unsupported research independently Would like to implement BID in the company Has many ideas Low to high knowledge about BID Contacts experts to help	1, 2, 6–10, 11, 17, 18
Project leader	Low to high knowledge about BID Is willing to test BID for specific purposes Knows what to use BID for in the company Does the project management	1, 2, 3, 6, 7, 9, 10, 11, 14, 15, 17, 18
CEO	Low to high knowledge about BID Needs to be convinced Sees long‐term opportunities Wants fast results Is willing to invest time and money, if the project sounds feasible and offers return on invest	2, 11, 18
Practitioner	Has extensive experience Has doubts, but is open minded Will support the project if it is not too much extra work	1, 2, 3, 5, 6, 7, 8, 10, 11, 13–17
Innovation/product manager	Wants to use BID for innovative new products Sees potential for unique selling points Uses BID for inspiration and first analogies No suitable project identified	1, 2, 6–10, 11, 15, 18
Curious employee	Low to high knowledge about BID First considerations to use BID Sees potential for innovation, new products and ideas No suitable project identified Has difficulties making a connection with own expertise	1, 2, 3, 4, 6, 7 ,9, 16, 17
Students		
Technical student	Is interested in the topic Wants to be inspired and to understand the process Thinks of future projects Has difficulties understanding biology in detail	1–18
Biology student	Studies biology with little focus on BID Is interested in the topic Has little awareness that fundamental knowledge from biology can serve technology Does not know how to transfer knowledge from biology to technology	1–18
Innovation management student	Is interested in the topic Wants to be inspired and to understand the process Has little interest in fundamental research Thinks of future innovation projects Has difficulties to understand biology in detail	1–18
Experts		
Expert (e.g., biologist, designer, architect, engineer)	Has a higher or trained education Wants to study and understand BID deeply Wants to transfer his/her knowledge to BID Has difficulties understanding the various approaches Wants to work in BID projects	1, 2, 6–18

The personas described in Table 4 indicate various needs and expectations toward the content and objectives of teaching and training. Therefore, we matched the personas with the learning objectives from Table 2. The survey with 20 students showed that objectives 3 (Use their own expertise for BID), objective 11 (Develop a biomimetic mindset), objective 12 (Develop their integrative skills), and objective 13 (Develop cognitive flexibility) were considered to be less relevant as they were mentioned by 20 or less percent of the students. However, we consider them to be of relevance and it might be due to the fact that students had had little experience with BID, as 90% of them considered their knowledge to be low to very low and had not yet begun to learn about biomimetics. Regarding the appropriateness of the personas, 45% considered the personas to be well or very well described, while 55% considered the personas to not fit perfectly as, for instance, they considered themselves to reflect a mix of described characteristics. We are well aware that additional aspects could be distinguished to further subcategorize these personas. For instance, the size of the company may be an important variable in integrating BID in the business environment. Small‐ and medium‐sized enterprises usually do not have specific departments for innovation management, and therefore, the role of a project manager might also be the innovation manager. Other personas must also exist, but it is difficult to identify and gather reliable data about them. For instance, there are ‘translational persona’ with the role of bridging the domains of biology and technology by bringing unique insights or methods to both. In addition, some objectives might be of relevance once a person holds another position. We consider these aspects to be a limitation of our analysis. However, persona describe a group of people, and as such, variations exist.

We could not identify curricula or teaching modules of BID‐targeting biology students in particular, except for a zoology course [85]. The ones identified were designed for engineering students [56, 67, 78] or for interdisciplinary courses in which both biology and engineering students can participate [73]. This indicates that the design of teaching modules for students studying biology is necessary, especially as they are essential in the process of BID [36, 38]. Engaging them more in BID could offer new (job) perspectives for biologists in the future, if they are well trained in the effective translation of biological information. In line with that, it was difficult to identify suitable curricula for experts with a degree who would like to learn more about BID and do not know how to use their fundamental knowledge for BID in application. Existing training is often linked to a single approach of BID, it is often very expensive, and as the approach is not suitable for every persona, no appropriate training exists so far. Therefore, BID should be integrated early in studying programs of biology and other BID‐related disciplines. Urmann [55], gives the most comprehensive overview of designing a curriculum in biomimicry.

### Tools used for steps of the process

The process of biomimetics can be supported by various tools and methods. In line with the analysis of the learning objectives and their inspirational or training capability, we analyzed which tools can be used for inspiration of users and which tools are relevant for teaching and training, as they require a deeper understanding and a detailed explanation within the context of the methodology.

As each of the 43 previously analyzed tools can be addressed to a certain step of the process [30], they can all be used for teaching and training and ultimately for the implementation of BID in practice. As inspirational tools, they can serve various types of innovation or product development. Many of the tools were developed for a specific persona and learning context (e.g., for teaching undergraduate students or for engaging industry practitioners). Some tools are being translated from one context to another and have met with varying degrees of success [98]. We consider more than half of the tools to be useful for teaching and training only, as they require deeper understanding which goes beyond inspirational information due to the time and knowledge needed to use them properly. In addition, most of the tools are not readily available, for example, as free online versions and therefore including them in the process seems difficult.

As the learning objectives are linked to inspiration or training, we identified which tools could facilitate the respective objectives. With regard to inspiration, only objective 5 (Discover and explore nature's patterns) and objective 14 (train creativity) can be matched directly with some tools, for instance AskNature [99]. The other objectives need to be understood in detail and required a deep understanding of tools and methods which could be used to achieve the learning outcomes. Yargin *et al*. [29], for instance, give a great insight in how to design analogical design tools in the context of BID, and Glier *et al*. [31] have already tested tools useful for teaching.

In most cases, no distinction of specific users and audiences of the tools is made. As most tools and methods refer to the process of biomimetics in general, the motivation for the respective developments was to overcome well‐known challenges and to support the tasks which need to be performed. For instance, to search for biological analogies does not differentiate between the users. So, in general, there was no audience analysis ahead of the development or it was not mentioned explicitly. However, when testing student settings, variation of accuracy in using a certain tool was tested with regard to, for example, disciplines, gender or class‐room settings [100].

Ongoing research will address the usability of tools in various contexts, for different audiences and in different learning settings.

## Conclusion

The aim of this manuscript was to clarify the state of the art, and identify gaps and needs in the context of teaching and training biomimetics. Based on the data presented, current and future curricula and training opportunities could be rethought and designed by (re)defining additional learning objectives, which address the description of personas and which could be aligned with respective tools and methods. Our results could also be linked with results from studies at individual institutions, for example, [72, 100, 101], to improve the respective existing curricula. We tested our own educational settings by checking whether our objectives are well aligned with our personas and whether we should revise our content. 11 out of the 18 presented learning objectives are part of our curricula, focusing on training rather than inspiration. We have taught BID to 311 engineering students and to 348 management students (innovation and product management, operations management), some of them being innovation managers in companies who study alongside their work. The learning objectives are, in general, well aligned with the personas; however, some objectives were missing. Therefore, we will pay particular attention to address the needs more accurately and we will revise the content of our curricula.

We could predict that tools would be used more often in training or practice if they were easily accessible and ready to use. On the other hand, we need to keep in mind that accessibility was not a key requirement for the consideration of tools for BID in practice, but rather BID‐specific requirements were mentioned [29]. In addition, most of the existing tools require training and deep understanding [2, 36]. This might be one of the drawbacks in BID, which could be addressed in the future by rethinking the design and contribution of tools and methods, having the audience in mind.

Students could be taught with an active learning teaching style using invention rather than the ‘tell and practice’ or just lecturing, as the deep understanding of the methodology needs a certain intensity and we did not identify any factual knowledge that is associated specifically with biomimetics as a process. Students could also be taught in the use of tools so that once they become a practitioner, they are able to use them effectively. Practitioners could focus on the tool kit and identify which tools are more appropriate for them. The training offered to them could be oriented along the learning objectives and it could happen in a more traditional educational style.

This manuscript raises many further research topics, in line with previously addressed open questions, for example, [50], like the roles of team members in ID teams, the intensity of teaching and training of tools in particular or the interdisciplinary collaboration. In addition, further research should investigate several assumptions in biomimetics, like the fundamental assumption that the required information for novel ideas is available in biology and just needs to be translated and transferred to technology. This manuscript has indicated that this transfer is way more challenging and complex than it might seem, and that future research in the addressed topics is necessary. However, we consider the overall findings to be helpful, in the very end, to increase the number of users who are not only excited and inspired but who are actually capable of implementing biomimetics in practice. This education of future users can be a step toward tapping the full potential of BID as other challenges, which need to be addressed with the transformation of the paradigm in BID into a scalable methodology, may benefit from progress in this field [11].

## Conflicts of interest

The authors declare no conflict of interest.

## Author contributions

KW performed the conceptualization; KW and SJ involved in the methodology; KW, SJ, and CZ involved in the validation; KW, DR, and SJ performed the formal analysis; KW, DR and SJ involved in the Investigation; DR and SJ performed the resources; KW performed the data curation; KW wrote original draft preparation; SJ and CZ wrote and reviewed the manuscript; KW, SJ, and CZ performed the visualization; SJ and CZ involved in the supervision.

## Data Availability

All data not subject to limited distribution due to confidentiality agreements are available upon reasonable request to the first author, KW.
